# Social and Poor Law Problems

**Published:** 1906-08-04

**Authors:** 


### SOCIAL AND POOR LAW PROBLEMS.

### WHAT IS A "NURSE"?

It is to be wished that Poor-law Unions, when advertising for a nurse, would state explicitly what they want and what they expect to get. Some Unions make it clear that they want a woman who has been trained for the work, while others frame their advertisements in such terms that any "single woman or widow without encumbrance" might apply with, apparently, a reasonable chance of getting the appointment. When, for example, the Epsom Union advertises for a "trained nurse," and adds that a knowledge of midwifery is essential, we know that the post is not meant for an entirely ignorant woman, though it would be better if it were clearly stated what is the minimum amount of training in nursing and midwifery required. The Eastry Union does better in stating that applicants for the post of nurse at their workhouse hospital "must possess the qualifications for superintendent nurses prescribed by the Local Government Board." This is a definite statement, which will keep the ranks of applicants from being swelled by a host of untrained and perhaps incapable women. The City of Norwich specifically demands a trained certificated nurse, and adds that candidates having the L.O.S. certificate for midwifery will be preferred. Advertisements couched in such language not only save trouble to the Guardians, but limit, if they do not altogether prevent, the chance of a local candidate being selected if she has not the requisite qualifications. But the same cannot be said for Bedford, which merely says that a certificated nurse will be preferred. We know how little such "preference" amounts to when set against the advantages of local influence. But what can be said for Worcester, which only announces that "the Guardians require a charge nurse for the Workhouse Infirmary." Not even an age-limit is mentioned, nor is training suggested. The term "charge nurse" might be taken to imply a person who has received some training, but we know that women without any training whatever are sometimes put in charge of sick paupers. Now that so many Poor-law Infirmaries give a full three years' training, there should be a supply of trained nurses to meet any demand. There is no longer an excuse for appointing a person who has not received a proper training to a position of responsibility, and if the Guardians of our Unions do not realise this it is time that pressure was brought to bear on them to limit their power of choice.

